# Interactions of six SNPs in APOA1 gene and types of obesity on low HDL-C disease in Xinjiang pastoral area of China

**DOI:** 10.1186/s12944-017-0581-8

**Published:** 2017-10-02

**Authors:** Xinping Wang, Jia He, Heng Guo, Lati Mu, Yunhua Hu, Jiaolong Ma, Yizhong Yan, Rulin Ma, Shugang Li, Yusong Ding, Mei Zhang, Qiang Niu, Jiaming Liu, Jingyu Zhang, Shuxia Guo

**Affiliations:** Department of Public Health and Key Laboratory of Xinjiang Endemic and Ethnic Diseases of the Ministry of Education, Shihezi University School of Medicine, Shihezi, 832000 China

**Keywords:** ApoA1, Polymorphism, Obesity, Interaction, Low HDL-C disease

## Abstract

**Background:**

This study aims to investigate association between six single nucleotide polymorphisms(SNPs) in APOA1 gene and types of obesity with the risk of low level HDL-C in the pastoral area of northwest China.

**Methods:**

A total of 1267 individuals including 424 patients with low HDL-C disease and 843 health subjects were analyzed based on matched for age, sex. SNPShot technique was used to detect the genotypes of rs670, rs5069, rs5072, rs7116797, rs2070665 and rs1799837 in APOA1 gene. The relationship between above six SNPs and types of obesity with low HDL-C disease was analyzed by binary logistic regression.

**Results:**

Carriers with rs670 G allele were more likely to get low HDL-C disease (OR = 1.46, OR95%CI: 1.118–1.915; *P* = 0.005); The genotypic and allelic frequencies of rs5069, rs5072, rs7116797, rs2070665, rs1799837 revealed no significant differences between cases and controls (*P* < 0.05); with reference to normal weight, Waist circumference (WC), Waist-to-hip ratio (WHR) individuals, respectively, general obesity measured by BMI had 2.686 times (OR95%CI: 1.695–4.256; *P* < 0.01), abdominal obesity measured by WC had 1.925 times (OR95%CI: 1.273–2.910; *P* = 0.002) and abdominal obesity measured by WHR had 1.640 times (OR95%CI: 1.114–2.416; *P* = 0.012) risk to get low HDL-C disease; APOA1 rs670 interacted with obesity (no matter general obesity or abdominal obesity) on low HDL-C disease.

**Conclusions:**

APOA1 gene may be associated with low HDL-C disease in the pastoral area of northwest China; obesity was the risk factor for low HDL-C disease; the low HDL-C disease is influenced by APOA1, obesity, and their interactions.

## Background

For years, the prevalence of Coronary Artery Disease (CAD) has been increasing. Many factors influence the risk to develop CAD, Low levels of high density lipoprotein cholesterol (HDL-C) is an independent traditional risk factor of CAD [1, 2]. APOA1 is the most abundant component of HDL-C which is required for normal HDL-C synthesis and its gene deletion results in extremely low HDL-C levels [3], The most important mechanism for cardioprotective effects of HDL-C against CAD is reverse transport of cholesterol from the peripheral tissues to the liver and its excretion in the bile [4].

The pathway of HDL-C metabolism involves genetic and environmental factors, Serum HDL-C level with up to 80% heritability has a strong genetic basis and variants detected up to now explain only 10% of HDL-C variations [5], Genetic variations and mutations in the APOA1 gene may reduce serum HDL-C levels [6, 7]. Among many environmental factors related to HDL-C, Obesity were most closely tied with serum low HDL-C level [8], At present, there are no universal diagnostic criteria for obesity around the world. Body mass index (BMI), waist circumference (WC), waist to hip ratio (WHR) are the most commonly used indicators.

The serum levels and function of HDL-C are influenced by genetic and environmental factors as well as their interactions [9]. Interactions between obesity and APOA1 polymorphisms may contribute towards diseases, rather than the individual effects of each factor. However, this hypothesis needs to be confirmed. The link between obesity and plasma lipid levels has been documented [10–13], nonetheless, the interactions of single nucleotide polymorphisms (SNPs) in ApoA1 gene and obesity on HDL-C level are limited. Here, we describe HDL-C profiles in a sample of aged 18 years and above among Xinjiang pastoral area of northwest China. Specifically, this study aims to investigate association between ApoA1 gene and different types of obesity with the risk of serum low HDL-C level which may explain the deleterious role of obesity on HDL-C level.

## Methods

### Study population

Using the group-matching method, 424 patients with low HDL-C disease were randomly selected as the case group and 843 normal subjects who had no evidence of diseases related to atherosclerosis, CAD, diabetes, liver diseases, renal diseases, or malignant tumors and were free from medications known to affect serum lipid levels were randomly selected as the control group from our previous stratified randomized cluster samples [14].

### Epidemiological survey and biochemical index detection

All study subjects completed a demographic information survey questionnaire during face-to-face interviews, Blood pressure, height, weight, waist circumference and hip circumference were measured according to standardized methods [15]. Laboratory analyses of blood samples included tests for fasting TG, LDL-C, TC, HDL-C and fasting plasma glucose (FPG) etc. all of which were analyzed using an automatic biochemical analyzer (AU400, Olympus: Tokyo, Japan). Various biochemical parameters were tested for all blood samples according to the 2007 China Adult Dyslipidemia Prevention Guide [16].

### Diagnostic criteria

BMI was calculated as weight in kilograms divided by the square of height in meters, according to the Chinese Working Group on Obesity [17], normal weight, overweight and obesity were defined as a BMI < 24, 24-28, and > 28 kg/m^2^; respectively. And a WC ≥ 85 cm in men or a WC ≥ 80 cm in women was considered representative of abdominal obesity, a WC < 85 cm in men or a WC < 80 cm in women was defined as normal WC [18, 19]. WHR was calculated as WC divided by the hip circumference, and a WHR ≥ 0.90 in men or a WHR ≥ 0.85 in women was classified as that representing abdominal obesity, a WHR < 0.90 in men or a WHR < 0.85 in women was classified as normal WHR [20].

Diagnostic criteria for low HDL-C disease: HDL-C < 1.04 mmol/L, Based on the China Adult Dyslipidemia Prevention Guide (2007) [16].

### DNA extraction

Fasting venous blood (200 μL) was taken from each study subject and a non-centrifugal columnar blood genomic DNA isolation kit (Tiangen, Beijing, China) was used to extract the whole blood genomic DNA. Extracted DNA was verified by gel electrophoresis (0.7% agarose). A NanoDrop spectrophotometer (NanoDrop technologies, Inc.: Wilmington, DE, USA) was used for quantitative determination of DNA concentration and purity: concentration ≥ 30 ng/μL and purity levels (OD260/OD280) of 1.7–2.0 were considered acceptable. Samples that met these criteria were diluted to 10–30 ng/μL using double-distilled water and stored at −80 °C until use.

### PCR amplification

Primers were designed using the Mysequenom tool (www.mysequenom.com/Home) and Assay Designer 3.0 software (SEQUENOM, Inc.: San Diego, CA, USA). Final PCR reaction volumes were 15 μL, which included 1 μL DNA samples, 0.3 μL dNTPs, 7.4 μLwater, 1.5 μL 10×PCR buffer, 1.5 μL MgCl_2_, 0.3 μLTaq enzymes, and 3 μL PCR amplification primer mixture. Cycling conditions were as follows: predegeneration at 94 °C for 4 min; followed by 35 cycles of denaturation at 94 °C for 20s, annealing at 56 °C for 30s, and extension at 72 °C for 1 min. A final extension step was carried out at 72 °C for 3 min, after which samples were maintained at 4 °C. Reactions were set up in an ice bath and each PCR experiment included a negative control reaction.

### PCR products purification

Shrimp alkaline phosphatase (SAP) was used to remove excess dNTPs from samples after PCR. This step served to ensure the accuracy of single-base extension. The final SAP reaction volumes were 5.0 mL, which included 0.5 μL 10 × SAP buffer, 2 μL PCR product, 2 μL double-distilled water, and 0.5 μL SAP enzyme. Reactions were carried out by incubation at 37 °C for 40 min, followed by incubation at 85 °C for 5 min. The reaction products were stored at 4 °C.

### Single-base extension

For single-base extension reactions, final reaction volumes were 6.0 μL, which included 0.5 μL Snapshot reagent, 2.5 μL water, 1 μL primer mix, 2 uL purified PCR products. Reaction conditions were as follows: denaturation at 94 °C for 30s; followed by 40 cycles of 94 °C for 5 s, 52 °C for 5 s, and finally 52 °C for 5 s. Reaction products were stored at 4 °C.

### Genotyping analysis

Take 1 μL reaction product plus 9 μL HIDI, 95 °C denaturation 3 min, immediately ice-water bath, all representative SNP genotyping experiments were done using TaqMan technology on an ABI3730XL system (Applied Biosystems: Carlsbad, CA, USA). T gene-mapper was used to complete the classification and output the results.

### Statistical analysis

Epidata 3.02 software (Epidata Association, Odense, Denmark) was used to establish a database, and the double entry method was used for data input and logic error detection. SPSS statistical software version 22.0 for Windows (IBM: Almon, NY, USA) was used for all data analysis. For descriptive statistics, *t*-test or Wilcoxon rank sum test were performed as appropriate after checking for normality, the chi-square test was used to evaluate differences between groups for the categorical variables. The gene counting method was used to calculate genotype and allele frequencies. The chi-square test was used to test for Hardy-Weinberg equilibrium, the odds ratio (OR) and 95% confidence interval (95% CI) and interactions between ApoA1 gene and types of obesity were assessed by binary logistic regression after controlling for potential confounders (sex, age, blood pressure, high TC, high TG, high LDL-C, smoker, drinker and FPG). *P* < 0.05 were considered statistically significant, the significance threshold was adjusted for multiple comparison tests according to Bonferroni correction, and set at *P* < 0.025 (0.05/2 = 0.025) when evaluating associations between genotypes in APOA1 gene and low HDL-C disease.

## Results

### Comparisons of general data of the subjects

Table 1 shows the general characteristics of the participants. In the case group, height, weight, waist circumference, waist-to-hip ratio, BMI, triglyceride, systolic blood pressure were higher than control group (*P* < 0.05), nevertheless, HDL-C, ApoA1, TC levels were lower than normal HDL-C group. There were no significant differences in the gender, age, LDL-C, FPG, diastolic blood pressure, smoker and drinker of each group (*P* > 0.05).

**Table 1:** The general characteristics between the control group and case group

Characteristics	Control(843)	Case(424)	*P* ^*a*^
Male/female	420/423	213/211	0.889
Age, years	44.35 ± 15.28	44.17 ± 15.05	0.805
Height, cm	161.10 ± 8.62	162.87 ± 9.28	0.001
Weight, kg	60.16 ± 11.65	67.76 ± 14.28	*P* < 0.001
Waist circumference, cm	83.29 ± 10.83	89.37 ± 12.19	*P* < 0.001
Waist-to-hip ratio	0.88 ± 0.08	0.91 ± 0.075	*P* < 0.001
Body mass index, kg/m2	23.11 ± 3.77	25.43 ± 4.27	*P* < 0.001
TC	4.56 ± 1.34	4.13 ± 1.16	*P* < 0.001
TG	1.15 ± 0.84	1.99 ± 1.59	*P* < 0.001
LDL-C	2.41 ± 0.97	2.26 ± 0.80	0.147
HDL-C	1.46 ± 0.33	0.87 ± 0.14	*P* < 0.001
Apolipoprotein A1 (g/l)	1.43 ± 0.27	1.05 ± 0.22	*P* < 0.001
FPG	4.65 ± 1.47	4.70 ± 1.76	0.640
Smoker, n (%)	211(25%)	112(26.4)	0.269
Alcohol drinker, n (%)	59(7%)	26(6.1)	0.321
Systolic blood pressure, mmHg	130.45 ± 23.62	132.55 ± 22.99	0.047
Diastolic blood pressure, mmHg	81.88 ± 14.12	83.29 ± 14.71	0.073

Values are presented either mean±SD or n (%). *a: t-test* or Wilcoxon rank sum test was used to obtained the *P* value for continuous variables, and a chi-square test was used to obtain significance for categorical variables, *P* < 0.05 significant

### Hardy-Weinberg equilibrium testing

In our study, six SNPs in the ApoA1 gene were genotyped, the success rates were all 100%. all loci were coincided with the Hardy-Weinberg equilibrium (*P* > 0.05), indicating that six loci of the ApoA1 gene reached genetic equilibrium and the samples were thus indeed representative of the pastoral area population of northwest China.

### Genotype and allele frequencies

The genotypic frequencies of six SNPs between normal and low HDL-C subjects are summarized in Table 2. the influence of nationalities, sex and age were eliminate by binary logistic regression analysis. For the rs670 polymorphism. Compared with A allele, rs670 G allele carrier had 1.46 times to get low HDL-C disease (OR95%CI: 1.118-1.915; *P* = 0.005). There were no significance differences in the genotype and allele frequencies of rs5069, rs5072, rs7116797, rs2070665 and rs1799837 SNPs between the normal and low HDL-C subjects (*P* > 0.05).

**Table 2:** The genotypic and allelic frequencies between the case and control group

SNP	Genetype/Allelic	Control	Case	OR^*a*^	95%CI	*P* ^*b*^
rs670	AA	12(1.4%)	16(3.8%)	1.00		
	AG	197(23.4%)	114(26.9)	2.62	1.059–6.465	0.037
	GG	634(75.2%)	294(69.3)	1.38	1.004–1.886	0.047
	G	1465(86.9%)	702(82.8%)	1.46	1.118–1.915	0.005
rs5069	CC	717(85.1)	364(85.8)	1.00		
	CT	126(14.9)	60(14.2%)	0.94	0.673–1.308	0.710
	T	132(7.8%)	60(7.1%)	1.12	0.813–1.532	0.500
rs5072	CC	416(49.3%)	207(48.8%)	1.00		
	CT	356(42.2%)	187(44.1%)	1.06	0.828–1.346	0.660
	TT	71(8.4%)	30(7.1%)	0.85	0.537–1.343	0.480
	T	498(29.5%)	247(29.1%)	1.02	0.851–1.223	0.830
rs7116797	AA	118(14.0%)	49(11.6%)	1.00		
	AG	393(46.6%)	210(49.5%)	1.29	0.886–1.868	0.190
	GG	332(39.4%)	165(38.9%)	1.20	0.817–1.753	0.360
	G	1057(62.7%)	540(63.7%)	0.96	0.808–1.138	0.630
rs2070665	CC	416(49.3%)	207(48.8%)	1.00		
	CT	355(42.1%)	187(44.1%)	1.06	0.530–1.350	0.650
	TT	72(8.5%)	30(7.1%)	0.84	0.530–1.323	0.450
	T	499(29.6%)	247(29.1%)	1.02	0.853–1.226	0.810
rs1799837	AG	11(1.3%)	4(0.9%)	1.00		
	GG	832(98.7%)	420(99.1%)	1.39	0.439–4.386	0.580
	G	1675(99.3%)	844(99.5%)	0.72	0.229–2.273	0.580

rs670-AA, rs5069-CC and rs5072-CC genotypes used as a reference genotype for obtaining the odds ratio calculations separated for each single nucleotide polymorphism

rs7116797-AA, rs2070665-CC and rs1799837-AG genotypes used as a reference genotype for obtaining the odds ratio calculations separated for each single nucleotide polymorphism

^*a, b*^Binary logistical regression analysis was used to obtained *P* value, the odds ratio (OR) and 95% confidence interval (95% CI) to obtained the SNPs difference between case and control group with adjustments for nationalities, age and sex. *P* < 0.025 significant

### Association of types of obesity with low HDL-C disease.

Association of different obesity types with low HDL-C disease are shown in Table 3. The risk degree was evaluated by low HDL-C (0 = no, 1 = yes) and analyzed by binary logistic regression analysis. After adjusting for confounding factors such as age, sex and nationalities, with reference to normal BMI, WC and WHR individuals, respectively, general obesity(BMI) had 2.686 times (OR95%CI: 1.695–4.256; *P* < 0.01), abdominal obesity (WC) had 1.925 times (OR95%CI: 1.273–2.910; *P* = 0.002) and abdominal obesity (WHR) had 1.640 times (OR95%CI: 1.114–2.416; *P* = 0.012) risk to get low HDL-C disease.

**Table 3:** Association of types of obesity with low HDL-C

Types of Obesity	*β*	SE	*Waldχ* ^2^	*P* ^*a*^	OR^*b*^	95%CI
General obesity (BMI)						
No	–	–	–	–	1	–
Yes	0.988	0.235	17.711	< 0.01	2.686	1.695–4.256
Abdominal obesity (WC)						
No	–	–	–	–	1	–
Yes	0.655	0.211	9.648	0.002	1.925	1.273–2.910
Abdominal obesity (WHR)						
No	–	–	–	–	1	–
Yes	0.495	0.198	6.271	0.012	1.640	1.114–2.416

^*a, b*^ *P* and OR values were obtained by binary logistical regression analysis with adjustments for age, sex, blood pressure, high TC, high TG, high LDL-C, smoker, drinker and FPG. *P* < 0.05 significant. General obesity(BMI): general obesity measured by BMI; Abdominal obesity (WC): abdominal obesity measured by WC; Abdominal obesity (WHR): abdominal obesity measured by WHR

### Interactions of rs670 and types of obesity on low HDL-C disease

The interactions of rs670 and types of obesity on low HDL-C disease are given in Table 4, The risk degree of interactions between obesity (normal BMI = 1, overweight = 2, general obesity = 3; normal WC = 0, abdominal obesity (WC) = 1; normal WHR = 0, abdominal obesity (WHR) = 1) and rs670 (AA = 1, AG = 2, GG = 3) was evaluated by low HDL-C disease (0 = no, 1 = yes) and analyzed by binary logistic regression analysis.

**Table 4:** Interactions of rs670 and types of obesity on Low HDL-C disease

Variable	*β*	SE	waldχ2	OR	95%CI	*P*
Normal weight*AA	–	–	–	1	–	–
General obesity(BMI)*AG	1.267	0.484	6.853	3.548	1.375–9.159	0.009
General obesity(BMI)*GG	1.232	0.292	17.862	3.428	1.936-6.071	< 0.001
Normal WC*AA	–	–	–	1	–	–
Abdominal obesity (WC)*AG	0.984	0.277	12.613	2.675	1.554–4.604	< 0.001
Abdominal obesity (WC)*GG	0.918	0.198	21.447	2.505	1.698–3.696	< 0.001
Normal WHR*AA	–	–	–	1	–	–
Abdominal obesity (WHR)*AG	0.735	0.278	6.997	2.086	1.210–3.597	0.008
Abdominal obesity (WHR)*GG	0.805	0.197	16.682	2.236	1.520–3.290	< 0.001

*Interactions between rs670 and different types of obesity, the interactions were assessed by binary logistic regression analysis after controlling for potential confounders (sex, age, blood pressure, high TC, high TG, high LDL-C, smoker, drinker and FPG). *P* < 0.025 significant

rs670 were shown interactions with obesity(no matter general obesity or abdominal obesity)on low HDL-C disease (*P* < 0.001–0.009). With reference to normal weight with rs670AA genotype individuals, the OR of general obesity with rs670AG genotype is 3.548 and that of general obesity with GG genotype is 3.428. Compared with normal WC with rs670AA genotype individuals, the ORs of abdominal obesity (WC) with AG and GG were 2.675 and 2.505. For abdominal obesity (WHR), the corresponding ORs were 2.086 and 2.236, as shown in Table 4.

## Discussion

Serum cholesterol levels are positively correlated with mortality of CAD [21]. Low level HDL-C is considered to be an independent risk factor for CAD occurs [22], the low HDL-C level has been described in several populations (Mexico [23]; Turkey [24], Thailand [25], India [26]; Iran [27]). However, the results are still inconsistent. Our previously results showed that the prevalence of low HDL-C among Xinjiang pastoral area were 33.6%, which was higher than the other phenotypes of dyslipidemia including elevated triglycerides, total cholesterol, and low-density lipoprotein cholesterol [28]. Plasma HDL-C level affected by many factors in human beings, the high prevalence of low HDL-C disease in Xinjiang pastoral area may be due to complex interactions between environmental and genetic factors.

In the current study, 424 low HDLC individuals and 843 normal individuals were analyzed, the level of BMI, WC, WHR were higher in cases than in normal individuals, whereas TC, HDL-C and APOA1 level was lower in cases, low HDL-C is usually associated with high levels of TC, in our study, the TC level of control subjects were higher than cases, may due to the primary foods in participants with low HDL-C levels contain high fat products such as wheat, beef, mutton, and dairy product consumption was higher than individuals with normal HDL-C levels [9].

In this study, the frequency of A allele in APOA1 gene rs670 loci was 0.130, lower than 0.320 in Taiwan population [29] and 0.18 in North America of Caucasian [30], but similar to 0.135 in Japan population [31], G allelic frequencies was 0.869 higher than 0.807 in Arab population [32]. The frequency of rs5069 C allele was 0.922 lower than 0.964 in Kuwait population [33], and rs5069 T allele was 0.078 notably higher than 0.020 in the Indians population that living in Singapore [34]. The C allelic frequency of rs5072 in this study was 0.705 higher than 0.630 in the population of Hyderabad, India [35]. Furthermore, The G allelic frequency of rs7116797 was 0. 627 lower than 0.667 of South Asian immigrant population [36]. Besides the T allelic frequency of rs2070665 was 0.296 in our population, higher than 0.222 than Yugu nationality but lower 0.294 than Han ethnic population in GanSu, northwest of China [37], in addition, the G allelic frequency of rs1799837 was 0.993 lower 0.997 in Arab population [32]. These results show that there exists significant racial/ethnic variation of allelic frequencies in the ApoA1 gene.

Numerous studies have been conducted on APOA1 rs670 polymorphism, nonetheless, the results still remain inconsistent. Haase et al. conducted a molecular analysis of the APOA1 gene found that rs670 was associated with increased HDL-C [38], however, S. Chhabra et al. have found that rs670 could serve as genetic factor of decreasing level of HDL-C in north Indian population [39], which consistent with the results of our study. But there are also studies have shown that rs670 A alleles gene mutations have no effect on HDL-C level [40–42]. In our current study, we did not detect any association between the rs5069, rs2070665, rs1799837, rs5072 and rs7116797 polymorphism and HDL-C levels, however, F Bandarian found that the association between rs2070665 with HDL-C levels are significant and rs2070665 were identified as independent predictors of HDL-C levels [43], inconsistent with our result. In addition, Elise [44] confirming the results that there were no association of the rs1799837 with lower plasma HDL-C levels, but Wang et al. found a significant association of HDL-C with rs1799837 [45]. Furthermore, Wu found that the rs5072 locus was associated with low HDL-C level [46], which was different from the results of this study, in another study, SE Hill observed that rs7116797 polymorphism was not associated with HDL-C levels [47], consistent with our results. These results suggest that there is association between ApoA1 gene and low HDL-C disease.

The potential association between SNPs and obesity in humans have been evaluated in several previous studies [48–50], but inconsistent in different populations. In this study, we found that there existed interactive effects between ApoA1 rs670 and obesity on low HDL-C diseases. Using normal individuals with rs670 AA genotype as reference in terms of BMI, WC, and WHR respectively, the ORs corresponding to rs670 AG and GG genotype were 3.548 and 3.428 for general obesity(BMI), 2.675 and 2.505 for abdominal obesity (WC), and 2.086 and 2.236 for abdominal obesity (WHR). These ORs were higher than those based on obesity type alone (2.686, 1.925 and 1.640). Therefore, the interactions between APOA1 rs670 and obesity might contribute to low HDL-C diseases, and might increase the risk of low HDL-C diseases. The results are in line with some previous studies [30, 50–52] which supports the association between ApoA1 gene, obesity and serum HDL-C levels.

Low HDL-C disease is influenced by multiple genes and environmental factors, each of which can contribute a minor marginal effect, conflicting results may result from studies that focused on the association of single gene and other risk factors with HDL-C levels, the contributions of gene-gene and gene-environment interactions to low HDL-C disease may provide a more mechanistic explanation for this condition.

## Conclusions

The current study found that there were no significant differences in the genotypic and allelic frequencies of APOA1 rs5069, rs5072, rs7116797, rs2070665, rs1799837 polymorphisms between normal HDL-C group and low HDL-C group, we also found that carriers with rs670 G allele were more likely to get low HDL-C disease. Besides, general obesity measured by BMI, abdominal obesity measured by WC, and abdominal obesity measured by WHR may serve as risk factors for low HDL-C disease; Finally, our results show that the low HDL-C disease is partly influenced by APOA1, obesity, and their interactions.

Admittedly, the present study has certain limitations, Firstly, the sample size in our studyis a bit small. Individuals with rs5069 TT genotype and rs1799837 AA genotype were not detected, Secondly, the HDL-C levels are affected by multiple environmental and genetic factors and their interactions, although we have discussed the interactions of six ApoA1 SNPs and obesity on low HDL-C disease, there are still many unclear environmental and genetic factors and their interactions that remain to be detected.

## Abbreviations

APOA1: Apolipoprotein A1
BMI: Body mass index
CAD: Coronary Artery Disease
FPG: Fasting plasma glucose
HDL-C: High-density lipoprotein-cholesterol
LDL-C: Low-density lipoprotein-cholesterol
SNPs: Single nucleotide polymorphisms
TC: Total cholesterol
TG: Triglyceride
WC: Waist circumference
WHR: Waist-to-hip ratio

## References

[1] Castelli WP (1988). Cholesterol and lipids in the risk of coronary artery disease--the Framingham Heart Study. Can J Cardiol.

[2] Ingelsson E, Schaefer EJ, Contois JH, JR MN, Sullivan L, Keyes MJ, Pencina MJ, Schoonmaker C, Wilson PW, Dag’ostino RB, Vasan RS (2007). Clinical Utility of Different Lipid Measures for Prediction of Coronary Heart Disease in Men and Women. JAMA.

[3] Rader DJ (2006). Molecular regulation of HDL metabolism and function: implications for novel therapies. J Clin Investig.

[4] Rye KA, Bursill CA, Lambert G, Tabet F, Barter PJ (2009). The metabolism and anti-atherogenic properties of HDL. J Lipid Res.

[5] Boes E, Coassin S, Kollerits B, Heid IM, Kronenberg F (2009). Genetic-epidemiological evidence on genes associated with HDL cholesterol levels: a systematic in-depth review. Exp Gerontol.

[6] Cho KH, Durbin DM, Jonas A (2001). Role of individual amino acids of apolipoprotein A-I in the activation of lecithin:cholesterol acyltransferase and in HDL rearrangements. J Lipid Res.

[7] Eckardstein AV (2006). Differential diagnosis of familial high density lipoprotein deficiency syndromes. Atherosclerosis.

[8] Liu XY, Lu Q, Lu WJ (2012). Recent advances in genetics research related to high density lipoprotein cholesterol levels. Biochem Biophys Adv.

[9] Yao MH, Guo H, Jia H, Yan YZ, Ma RL, Ding YS, Zhang JY, Liu JM, Mei Z, Li SG (2016). Interactions of Six SNPs in ABCA1gene and Obesity in Low HDL-C Disease in Kazakh of China. Int J Environ Res Public Health.

[10] Yin RX, Wu DF, Miao L, Htet Aung LH, Cao XL, Yan TT, Long XJ, Liu WY, Zhang L, Li M (2013). Interactions of several single nucleotide polymorphisms and high body mass index on serum lipid traits. Biofactors.

[11] Askari G, Heidari-Beni M, Mansourian M, Esmaeil-Motlagh M, Kelishadi R (2016). Interaction of lipoprotein lipase polymorphisms with body mass index and birth weight to modulate lipid profiles in children and adolescents: the CASPIAN-III Study. Sao Paulo Med J Rev Paul Med.

[12] Heidaribeni M, Kelishadi R, Mansourian M, Askari G (2015). Interaction of cholesterol ester transfer protein polymorphisms, body mass index, and birth weight with the risk of dyslipidemia in children and adolescents: the CASPIAN-III study. Iran J Basic Med Sci.

[13] Song YY, Gong RR, Zhang Z, Li YH, Fan M, Ou GJ, Fang DZ (2014). Effects of Apolipoprotein A1 Gene rs670 and rs5069 Polymorphisms on the Plasma Lipid Profiles in Healthy Adolescents with Different Body Mass Index. Zhongguo Yi Xue Ke Xue Yuan Xue Bao Acta Academiae Medicinae Sinicae.

[14] He J, Guo S, Liu J, Zhang M, Ding Y, Zhang J, Li S, Xu S, Niu Q, Guo H (2014). Ethnic differences in prevalence of general obesity and abdominal obesity among low-income rural Kazakh and Uyghur adults in far western China and implications in preventive public health. PLoS One.

[15] Series WHOTR (2000). Obesity: preventing and managing the global epidemic. Report of a WHO consultation. World Health Organ Tech Rep.

[16] Prevention JCfDCgo, Adults ToDi (2007). Chinese guidelines on prevention and treatment of dyslipidemia in adults. Zhonghua Xin Xue Guan Bing Za Zhi.

[17] Zhou BF (2002). Predictive Values of Body Mass Index and Waist Circumference for Rish Factors of Certain Related Diseases in Chinese Adults—Study on Optimal Cut—off Points of Body Mass Index and Waist Circumference. BES.

[18] Chen C, Lu FC (2004). The guidelines for prevention and control of overweight and obesity in Chinese adults. Biom Environ Sci.

[19] Zhou B (2002). Predictive values of body mass index and waist circumference to risk factors of related diseases in Chinese adult population. Zhonghua Liu Xing Bing Xue Za Zhi.

[20] Who W, World Bank W: Obesity: Preventing and Managing the Global Epidemic. Report of WHO Consultation on Obesity. 1998. 11234459

[21] Martin MJ, Hulley SB, Browner WS, Kuller LH, Wentworth D (1986). Serum cholesterol, blood pressure, and mortality: implications from a cohort of 361,662 men. Lancet.

[22] Jr GA, Brinton EA (2004). Assessing low levels of high-density lipoprotein cholesterol as a risk factor in coronary heart disease: a working group report and update. J Am Coll Cardiol.

[23] Aguilar-Salinas CA, Olaiz G, Valles V, Torres JM, Gómez Pérez FJ, Rull JA, Rojas R, Franco A, Sepulveda J (2001). High prevalence of low HDL cholesterol concentrations and mixed hyperlipidemia in a Mexican nationwide survey. J Lipid Res.

[24] Adam B, Talu C, Bedir A, Alvur M, Sağkan O (1999). The levels of lipids, lipoproteins and apolipoproteins in healthy people in the central region of the Black Sea. Jpn Heart J.

[25] Pongchaiyakul C, Hongsprabhas P, Pisprasert V, Pongchaiyakul C (2006). Rural-urban difference in lipid levels and prevalence of dyslipidemia: a population-based study in Khon Kaen province, Thailand. J Med Assoc Thail.

[26] Mulukutla SR, Venkitachalam L, Marroquin OC, Kip KE, Aiyer A, Edmundowicz D, Ganesh S, Varghese R, Reis SE (2008). Population variations in atherogenic dyslipidemia: A report from the HeartSCORE and IndiaSCORE Studies. J Clin Lipidol.

[27] Sharifi F, Mousavinasab SN, Soruri R, Saeini M, Dinmohammadi M (2008). High prevalence of low high-density lipoprotein cholesterol concentrations and other dyslipidemic phenotypes in an Iranian population. Metab Syndr Relat Disord.

[28] Li YP, Ma RL, Zhang M, Liu JM, Ding YS, Guo H, Zhang JY, Li SG, Sun F, Guo SX (2013). Epidemic features of dyslipidemia among Uygur, Kazakh, and Han adults in Xinjiang, China in 2010. Zhonghua yu fang yi xue za zhi [Chinese journal of preventive medicine].

[29] Wu JH, Kao JT, Wen MS, Lo SK (2000). DNA polymorphisms at the apolipoprotein A1-CIII loci in Taiwanese: correlation of plasma APOCIII with triglyceride level and body mass index. J Formos Med Assoc.

[30] Chen ES, Mazzotti DR, Furuya TK, Cendoroglo MS, Ramos LR, Araujo LQ, Burbano RR, De ACSM (2009). Apolipoprotein A1 gene polymorphisms as risk factors for hypertension and obesity. Clin Exp Med.

[31] Bai H, Saku K, Liu R, Jimi S, Arakawa K (1996). Analysis of a new polymorphism in the human apolipoprotein A-I gene: association with serum lipoprotein levels and coronary heart disease. J Cardiol.

[32] Al-Bustan SA, Al-Serri AE, Annice BG, Alnaqeeb MA, Ebrahim GA (2013). Re-sequencing of the APOAI promoter region and the genetic association of the −75G > A polymorphism with increased cholesterol and low density lipoprotein levels among a sample of the Kuwaiti population. BMC Med Genet.

[33] Al-Bustan SA, Al-Serri AE, Annice BG, Alnaqeeb MA, Ebrahim GA (2013). Re-sequencing of the APOAI promoter region and the genetic association of the -75G > A polymorphism with increased cholesterol and low density lipoprotein levels among a sample of the Kuwaiti population. BMC Med Genet.

[34] Al-Yahyaee SA, Al-Kindi MN, Al-Bahrani AI (2004). Apolipoprotein A1 gene polymorphisms at the -75 bp and +83/ 84 bp polymorphic sites in healthy Omanis compared with world populations. Hum Biol.

[35] Pranavchand R, Kumar AS, Reddy BM (2017). Genetic determinants of clinical heterogeneity of the coronary artery disease in the population of Hyderabad, India. Human Genomics.

[36] Dodani S, Dong Y, Zhu H, George V (2008). Can novel Apo A-I polymorphisms be responsible for low HDL in South Asian immigrants?. Ind J Hum Genet.

[37] Gu QL, Lan YM, Zhu JD. Association of rs2070665 polymorphism of apolipoprotein B gene with lipid levels in Yugur and Han population of Sunan. J Clin Cardiol. 2015;751–4. http://xueshu.baidu.com/s?wd=paperuri%3A%283a54fdea9cd2e8fae32f73145bdcd78d%29&filter=sc_long_sign&tn=SE_xueshusource_2kduw22v&sc_vurl=http%3A%2F%2Fkns.cnki.net%2FKCMS%2Fdetail%2Fdetail.asp8&sc_us=13617646101411991505.

[38] Haase CL, Tybjærg-Hansen A, Grande P, Frikke-Schmidt R (2010). Genetically elevated apolipoprotein A-I, high-density lipoprotein cholesterol levels, and risk of ischemic heart disease. J Clin Endocrinol Metab.

[39] Chhabra S, Narang R, Lakshmy R, Das N (2004). APOA1-75 G to A substitution associated with severe forms of CAD, lower levels of HDL and apoA-I among northern Indians. Dis Markers.

[40] Akita H, Chiba H, Tsuji M, Hui SP, Takahashi Y, Matsuno K, Kobayashi K (1995). Evaluation of G-to-A substitution in the apolipoprotein A-I gene promoter as a determinant of high-density lipoprotein cholesterol level in subjects with and without cholesteryl ester transfer protein deficiency. Hum Genet.

[41] Smith JD, Brinton EA, Breslow JL (1992). Polymorphism in the human apolipoprotein A-I gene promoter region. Association of the minor allele with decreased production rate in vivo and promoter activity in vitro. J Clin Investig.

[42] Needham EWA, Mattu RK, Rees A, Stocks J, Galton DJ (1994). A Polymorphism in the Human Apolipoprotein AI Promoter Region: A Study in Hypertriglyceridaemic Patients. Hum Hered.

[43] Bandarian F, Hedayati M, Daneshpour MS, Naseri M, Azizi F (2013). Genetic Polymorphisms in the APOA1 Gene and their Relationship with Serum HDL Cholesterol Levels. Lipids.

[44] Villard EF, Ei KP, Frisdal E, Bruckert E, Clement K, Bonnefontrousselot D, Bittar R, Le GW, Guerin M (2013). Genetic determination of plasma cholesterol efflux capacity is gender-specific and independent of HDL-cholesterol levels. Arterioscler Thromb Vasc Biol.

[45] Wang XL, Badenhop R, Humphrey KE, Wilcken DE (1996). New MspI polymorphism at +83 bp of the human apolipoprotein AI gene: association with increased circulating high density lipoprotein cholesterol levels. Genet Epidemiol.

[46] Wu YH. Study on the interaction between environmental factors of metabolic syndrome and APOA1-APOC3-APOA4-APOA5 gene cluster polymorphism. Jilin: Jilin University of China; 2015.

[47] Hill SE. Sequence Variation in the APOA1 and APOA4 Genes and their Relationship with Plasma HDL-Cholesterol Levels. Arizona: University of Pittsburgh; 2009.

[48] Chen ES, Furuya TK, Mazzotti DR, Ota VK, Cendoroglo MS, Ramos LR, Araujo LQ, Burbano RR, De ACSM. APOA1/A5 variants and haplotypes as a risk factor for obesity and better lipid profiles in a Brazilian Elderly Cohort. Lipids. 2010;45:511–7. 10.1007/s11745-010-3426-z 20480398

[49] Min L, Lu Q, Yong Z, Gang T (2011). ApoB/apoA1 is an effective predictor of coronary heart disease risk in overweight and obesity. J Biomed Res.

[50] Toptas B, Görmüş U, Ergen A, Gürkan H, Keleşoglu F, Darendeliler F, Bas F, Dalan AB, Izbirak G, Isbir T (2011). Comparison of lipid profiles with APOA1 MspI polymorphism in obese children with hyperlipidemia. Vivo.

[51] Ma YQ, Thomas GN, Ng MC, Critchley JA, Cockram CS, Chan JC, Tomlinson B (2003). Association of two apolipoprotein A-I gene MspI polymorphisms with high density lipoprotein (HDL)-cholesterol levels and indices of obesity in selected healthy Chinese subjects and in patients with early-onset type 2 diabetes. Clin Endocrinol.

[52] Hassan NE, El-Masry SA, Zarouk WA, Elneam AIA, Rasheed EA, Mahmoud MM (2015). Apolipoprotein B polymorphism distribution among a sample of obese Egyptian females with visceral obesity and its influence on lipid profile. Genet Eng Biotechnol J.

